# 2014 *JRR* Terashima Award

**DOI:** 10.1093/jrr/rru103

**Published:** 2014-11

## Comment from the Editor-in-Chief


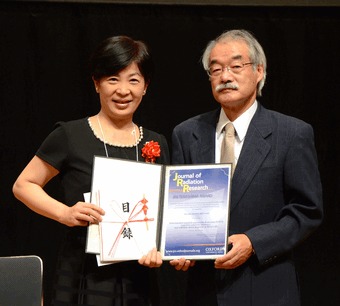


The Board of Editors of the *Journal of Radiation Research* is pleased to announce that Dr. Qiu-Mei ZHANG-AKIYAMA and co-authors are the winners of the 2014 Terashima Award, given for their outstanding paper published in the *Journal of Radiation Research* in 2012 and 2013. In 2001, the *Journal of Radiation Research* established the Terashima Award honoring Dr. Toyozo Terashima, a pioneer in Japan in the field of radiation research, and has since been presented to the most prominent paper each year. In 2014, five papers were nominated for the Award and one paper by Dr. ZHANG-AKIYAMA et al. received the highest evaluation. She and the co-authors received a certificate, a 100,000 yen award, and exemption from processing charge on their next article published in the *Journal of Radiation Research*.

## Comment from the Author

It is a great honor for us to receive the Terashima Award for our latest paper published in JRR. Reactive oxygen species (ROS) act as a mediator of ionizing radiation-induced cellular damage. Previous studies have indicated that Mn-SOD (SOD2) plays a critical role in protection against ionizing radiation in mammalian cells. However, the underlying mechanism is still not clear. In this study, we constructed stable HeLa cell lines overexpressing SOD2, to elucidate the mechanism underlying protection against radiation by SOD2. The overexpression of SOD2 in the mitochondria markedly lowered the levels of ROS in the cytoplasm and nucleus in irradiated cells. SOD2-expressing cells showed suppression of superoxide generation in mitochondria. Furthermore, irradiated SOD2-overexpressing cells exhibited a larger decrease in phosphorylated histone γ-H2AX level compared with irradiated control HeLa cells. DNA microarray analysis revealed different expression profiles in irradiated control cells and irradiated SOD2-overexpressing cells. These results indicate that SOD2 protects cells against γ-rays through suppressing ROS generated in mitochondria and regulating the expression of genes, which play critical roles in protection against ionizing radiation. We are delighted that our study has been widely accepted in our research field and frequently cited.

